# Oncologic Therapies

**DOI:** 10.1038/sj.bjc.6601671

**Published:** 2004-03-16

**Authors:** S J Strauss

**Affiliations:** 1 Cancer Research UK, Department of Medical Oncology, St Bartholomew's Hospital, London

The stated goal of the second edition of this text is to ‘provide a brief introduction to the principles and practice of oncology’, the book being aimed at junior doctors with an interest in oncology and junior oncologists ‘seeking a brief and focused patient management resource’. This is not an inconsiderable task and I found these goals were met in a very accessible and readable textbook

The book is divided into four parts with part one providing an introduction to the major therapeutic modalities of cancer management and the principles behind them. The chapters were well presented and informative. I found the section on chemotherapeutic agents and their mechanisms of action particularly helpful and summarised in a useful manner, ending with a table that provides for easy reference. Also included in this section was a chapter on the development of new therapies, and some of the ethical issues surrounding clinical trials, equipoise and the complex issues involved in gaining informed consent for treatment. As a substantial proportion of cancer therapy is based on research protocols, this subject is relevant to many junior staff who enter patients into clinical trials and seek informed consent. This chapter is thus very useful for gaining a better understanding of these issues that are critical to good clinical practice.

Part II is an introduction to the complications of malignancy and its treatment, and provides a brief overview of supportive care. This section was somewhat biased to a North American readership. For example the National Comprehensive Cancer Network (NCCN) guidelines were quoted for pain management, which are not used in the United Kingdom. Nonetheless the information and algorithms were helpful without being prescriptive. The chapter on chemotherapy-induced emesis was well written and again the use of comprehensive tables for the assessment of risk of emesis will be a helpful guide to a junior doctor. I was also pleased to note the inclusion of a chapter on quality of life, which is increasingly recognised as an important aspect of patient management and measure of treatment outcome. I was disappointed, however, at the exclusion of any information on the late effects and complications of therapy, as this too is important as the number of long-term survivors increases.

Part III describes the management of specific haematological malignancies and part IV describes solid tumours with chapters on specific tumour sites. These chapters were well organised and the standardised format was helpful as a resource, although the amount of detail varied between chapters. For example, the chapter on ovarian cancer was perhaps lacking in detail particularly in relation to the treatment of recurrent disease. Unfortunately, there was little or no use of pictorial or graphical representation and this was particularly missed in the haematological chapters where histopathological sections of peripheral blood, bone marrow or tissue biopsies would have been useful. The information was generally up to date and gave a good evidence-base for current management strategies. I found the chapters with tables of current data and trial results were more informative than those without. There was no use of Kaplan–Meier survival curves, which may have been a deliberate decision, however, their exclusion removed an easily accessible representation of comparative and long-term data. Overall, the reference lists were helpful and comprehensive enough to enable further reading for those interested in specific subjects. The indexing was however a little confusing and incomplete at times.

One noticeable omission from the introductory part of the book was a chapter about the genetic and molecular basis of cancer, which already provides valuable insights into the pathogenesis of many cancer types and also provides important prognostic information. New technologies for investigation of malignancy such as the use of gene expression profiling did certainly warrant a description. In addition, a more general analysis of the future of oncology and the basis for the development of new therapeutic agents would have been interesting.

Despite these reservations, I found Dr Vokes and Dr Golomb's book readable and useful as a primary resource. I would recommend this as a text for a dedicated junior oncologist, but feel it is pricey for a clinician with an ‘interest in oncology’.

